# A Tale of Two Ps: Panniculitis Secondary to Acute Pancreatitis

**DOI:** 10.7759/cureus.20504

**Published:** 2021-12-18

**Authors:** Vijairam Selvaraj, Kwame Dapaah-Afriyie

**Affiliations:** 1 Internal Medicine, Miriam Hospital, Providence, USA; 2 Medicine, Brown University, Providence, USA; 3 Hospital Medicine, Miriam Hospital, Providence, USA

**Keywords:** pancreatic disease, lipase, erythema nodosum, pancreatitis, panniculitis, lobular panniculitis

## Abstract

Pancreatic panniculitis (PP) is a rare variant of panniculitis that affects patients with pancreatic disorders, most commonly pancreatic malignancy or acute/chronic pancreatitis. Patients often present with painful, erythematous nodules on their lower extremities that may undergo spontaneous ulceration and necrosis. Treatment is largely supportive and should address the underlying pancreatic disease.

## Introduction

Pancreatic disorders, such as acute/chronic pancreatitis or pancreatic malignancy, may be infrequently accompanied or preceded by panniculitis, occasionally even causing ulceration and fistulation of the necrotic fat to the skin. Pancreatic panniculitis (PP) is a rare entity and affects only 0.3-3% of patients across a range of different pancreatic disorders [1]. In 40% of the cases, PP has been seen to precede pancreatic disease by several days to months. In this report, we describe a unique case of an elderly female who presented with non-healing lesions of the lower extremities despite treatment with multiple antibiotics and was found to have PP.

## Case presentation

A 65-year-old female with a history of limited scleroderma and primary biliary cholangitis (PBC) was admitted due to a rash in the bilateral lower legs. The rash had first manifested approximately one month ago as tender, red nodules in the bilateral lower legs. It had initially started after the patient had visited her family in Pennsylvania. Five days later, her primary care provider had prescribed cephalexin although this had been subsequently discontinued as the rash had worsened after starting the antibiotic. Three days later, she had been seen by dermatology who had started her on prednisone and doxycycline. A punch biopsy performed at that time had shown predominantly septal panniculitis with neutrophils, eosinophils, and septal fibrosis with a histologic pattern compatible with mid-to-late-stage erythema nodosum (EN). Antinuclear antibodies (ANAs) were observed to be elevated at 1:2560 (normal level: <1:40) with a centromere pattern, consistent with her known diagnosis of scleroderma. Antistreptolysin O, hepatitis C Ab, hepatitis B antigens, antineutrophil cytoplasmic antibodies (ANCA) screen, and QuantiFERON-TB Gold were negative. She normally took aspirin, gabapentin, pravastatin, ursodiol, and omeprazole at home; however, the EN was not thought to be drug-induced.

Two weeks later, she had followed up at the dermatologist's office where a culture of the purulent lesion on her right lower extremity was taken. The culture grew *Pseudomonas aeruginosa* for which she was started on ciprofloxacin. Four days later, she had developed an ulcerative lesion of the right lower extremity. She had been referred to the hospital from the dermatologist's office due to concern for infection and the need for intravenous antibiotics for cellulitis secondary to *Pseudomonas aeruginosa*. She also reported malaise, arthralgia, and a 10-pound weight loss over one month. She denied having any abdominal pain or gastrointestinal symptoms. She reported drinking three glasses of wine daily.

**Figure 1:**
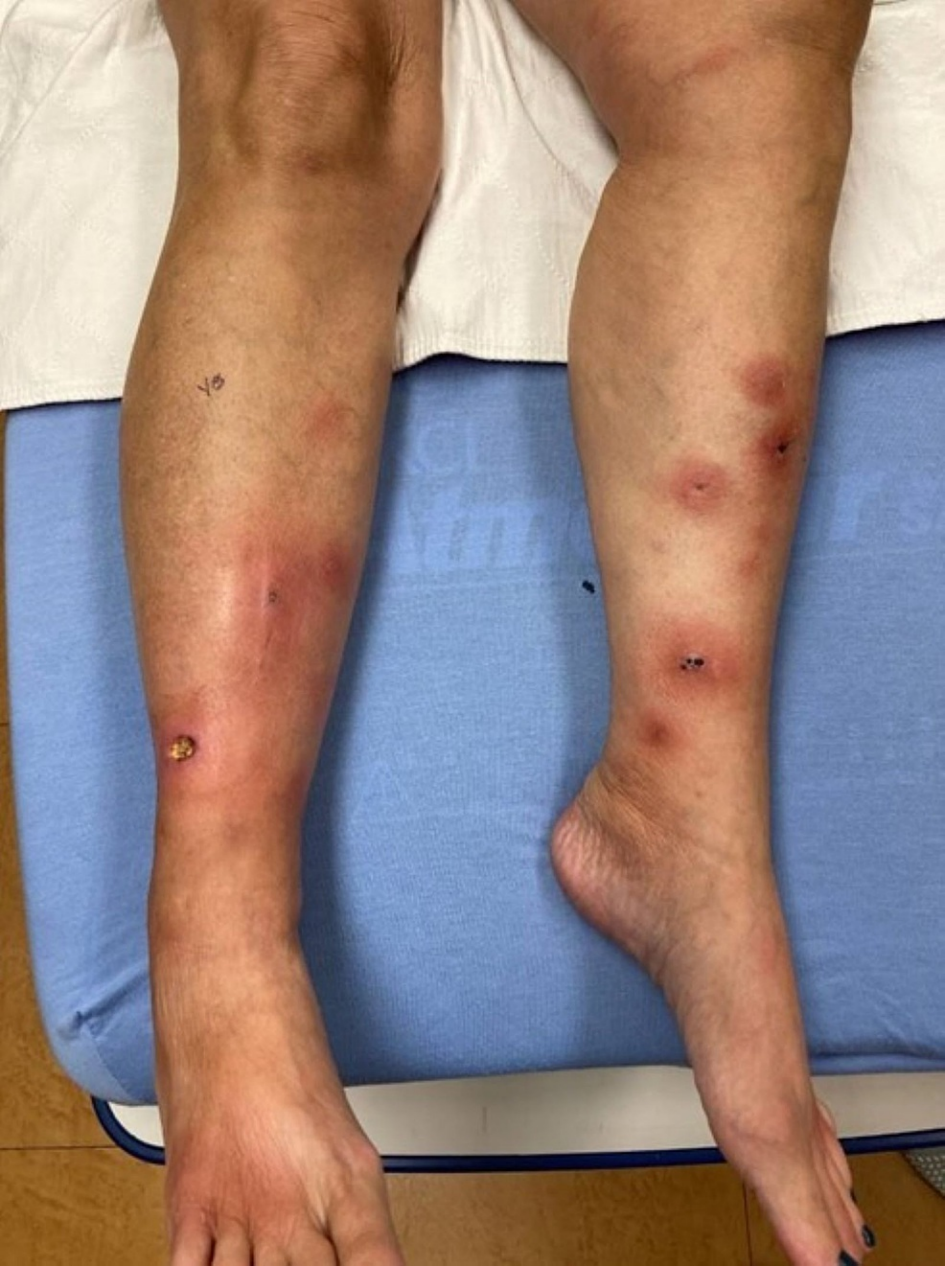
Image of bilateral legs showing numerous edematous pink plaques, some with central ulcerative eschar

On admission, her temperature was 98.1 °F, heart rate was 102 beats/minute, blood pressure was 130/66 mmHg, and her respiratory rate was 16 breaths/minute. She was saturating at 98% on ambient air. Her physical exam was remarkable for left and right shin with numerous edematous pink plaques, some with central ulcerative eschar (Figure 1). She also had bilateral hips/lateral proximal upper thighs with more mild edematous pink plaques. Labs were remarkable for a WBC count of 16300 cells/µL (normal range: 3500-11000 cells/µL), hemoglobin of 9.9 g/dl (normal range: 11-15 g/dl), lipase of >6000 IU/L (normal range: 10-60 IU/L), amylase of 1736 U/L (normal range: 28-100 U/L) and CA 19-9 of 72.2 U/mL (normal range: 0-35 U/mL). Triglyceride level was 60 mg/dl (normal range: 40-149 mg/dl), and c-reactive protein (CRP) was 27.70 mg/L (normal range: 0-10 mg/L). She was initially started on intravenous piperacillin-tazobactam every six hours, intravenous fluids, and ketorolac for pain management. On day one of her hospitalization, dermatology was consulted, and they recommended applying vaseline to the affected lesions and advised working her up further for the etiology of pancreatitis.

Given her history of limited scleroderma, PBC, and elevated pancreatic enzymes, an MRI of the abdomen was performed on day three, which showed nonspecific hepatic inflammation and probable groove pancreatitis with a dedicated pancreatic pseudocyst. She was continued on intravenous fluids, supportive care, and antibiotics. Again, given her history of scleroderma and PBC, autoimmune pancreatitis was considered; however, her IgG4 level was normal [55 mg/dl (normal range: 1-123 mg/dl)]. Gastroenterology was consulted, and they recommended endoscopic ultrasound on an outpatient basis and repeat MRI in eight weeks. On day four of her hospital course, her lipase decreased to 1615 IU/L and she was discharged home on oral ciprofloxacin and ibuprofen. Two weeks after discharge, she reported that her rash was improving and her wounds were healing well. Her lipase and amylase levels had decreased further to 321 IU/L and 233 U/L, respectively.

## Discussion

PP is the inflammation of the subcutaneous tissue associated with pancreatic disease. Main pancreatic diseases associated with PP are acute or chronic pancreatitis, pancreatic carcinoma, and intraductal papillary mucinous neoplasm. Most patients present with firm nodules or plaques on the lower limbs. There may also be erythematous, suppurative lesions with viscous liquid due to fat liquefaction. Lesions may also spread to the buttocks, trunk, arms, and scalp and can occur around joints and lead to arthritis. The triad of pancreatitis, panniculitis, and polyarthritis is sometimes known as PPP syndrome [2].

The exact cause of PP is unclear. Several mechanisms have been implicated in its pathogenesis. Firstly, the release of pancreatic enzymes such as lipase and amylase is believed to be the essential causative factor. Trypsin is thought to increase the permeability of the microcirculation and lymphatic channels, allowing lipase and amylase to enter the peripheral circulation. These enzymes promote lipolysis, resulting in glycerol and free fatty acid accumulation leading to adipocyte necrosis and panniculitis. Elevated enzyme levels can occur even in the absence of a detectable pancreatic disease [3]. Secondly, vascular damage and deposition of immune complexes may play a role in PP. Lastly, it may also be related to the release of adipokines. PP associated with pancreatic cancer tends to ulcerate, recur, and persist compared to inflammatory conditions.

PP is usually diagnosed by skin biopsy. Histologically, there are two types of PP: septal panniculitis and lobular panniculitis. Usually, it presents as lobular panniculitis without evidence of vasculitis. However, in the early stages, it may manifest as septal panniculitis due to the enzymatic damage of the septa, allowing pancreatic enzymes to cross from the blood to fat lobules resulting in necrosis of the adipocytes. The hallmark finding of PP is the presence of subcutaneous lobular fat necrosis with anuclear adipocytes (ghost adipocytes) surrounded by an inflammatory infiltrate [4,5]. The most common form of panniculitis is EN. Differentials often include erythema induratum, lupus panniculitis, or α1-antitrypsin-deficiency panniculitis [6].

Treatment is primarily supportive and should address the underlying pancreatic disease [5,7]. Lesions heal once the primary pathology is addressed. Prognosis typically depends on the underlying pathology and is often poor in cases associated with pancreatic carcinoma. The condition can be associated with a high mortality risk unless the primary pathology is resolved.

## Conclusions

In conclusion, the diagnosis of PP may be challenging given the absence of abdominal symptoms. Unrecognized pancreatic diseases may prove to be fatal. Clinicians must be aware that panniculitis may be the warning signal of serious illnesses such as pancreatic cancer, and may precede the signs and symptoms typically related to such illnesses. Extra attention should be given when panniculitis is persistent, and frequent relapses or ulceration of the nodules are regarded as red flags. Early biopsy and treatment of the underlying pathology are essential to improve symptoms and avoid inappropriate testing and risky medications.
